# Preclinical evaluation of novel fatty acid synthase inhibitors in primary colorectal cancer cells and a patient-derived xenograft model of colorectal cancer

**DOI:** 10.18632/oncotarget.25361

**Published:** 2018-05-15

**Authors:** Yekaterina Y. Zaytseva, Piotr G. Rychahou, Anh-Thu Le, Timothy L. Scott, Robert M. Flight, Ji Tae Kim, Jennifer Harris, Jinpeng Liu, Chi Wang, Andrew J. Morris, Theru A. Sivakumaran, Teresa Fan, Hunter Moseley, Tianyan Gao, Eun Y. Lee, Heidi L. Weiss, Timothy S. Heuer, George Kemble, Mark Evers

**Affiliations:** ^1^ Department of Toxicology and Cancer Biology, University of Kentucky, Lexington, KY, USA; ^2^ Markey Cancer Center, University of Kentucky, Lexington, KY, USA; ^3^ Department of Surgery, University of Kentucky, Lexington, KY, USA; ^4^ RC-SIRM, University of Kentucky, Lexington, KY, USA; ^5^ Cardiovascular Research, University of Kentucky, Lexington, KY, USA; ^6^ Department of Pathology and Laboratory Medicine, University of Kentucky, Lexington, KY, USA; ^7^ 3-V Biosciences, Menlo Park, CA, USA

**Keywords:** colorectal cancer, FASN, lipogenesis, patient-derived xenografts, TVB-3664

## Abstract

Fatty Acid Synthase (FASN), a key enzyme of *de novo* lipogenesis, is upregulated in many cancers including colorectal cancer (CRC); increased FASN expression is associated with poor prognosis. Potent FASN inhibitors (TVBs) developed by 3-V Biosciences demonstrate anti-tumor activity *in vitro* and *in vivo* and a favorable tolerability profile in a Phase I clinical trial.

However, CRC characteristics associated with responsiveness to FASN inhibition are not fully understood. We evaluated the effect of TVB-3664 on tumor growth in nine CRC patient-derived xenografts (PDXs) and investigated molecular and metabolic changes associated with CRC responsiveness to FASN inhibition.

CRC cells and PDXs showed a wide range of sensitivity to FASN inhibition. TVB-3664 treatment showed significant response (reduced tumor volume) in 30% of cases. Anti-tumor effect of TVB-3664 was associated with a significant decrease in a pool of adenine nucleotides and alterations in lipid composition including a significant reduction in fatty acids and phospholipids and an increase in lactosylceramide and sphingomyelin in PDXs sensitive to FASN inhibition. Moreover, Akt, Erk1/2 and AMPK were major oncogenic pathways altered by TVBs.

In summary, we demonstrated that novel TVB inhibitors show anti-tumor activity in CRC and this activity is associated with a decrease in activation of Akt and Erk1/2 oncogenic pathways and significant alteration of lipid composition of tumors. Further understanding of genetic and metabolic characteristics of tumors susceptible to FASN inhibition may enable patient selection and personalized medicine approaches in CRC.

## INTRODUCTION

Colorectal cancer (CRC) is the 2nd most common cause of cancer death in the United States [1]. Despite advances in our understanding of the molecular basis of CRC and the increasing number of targeted therapies, treatment frequently remains disappointing, particularly for advanced stages of disease [2, 3]. New approaches to treat advanced CRC are urgently needed to improve clinical outcomes.

Metabolic reprogramming is a hallmark of cancer, controlling various aspects of malignant development and progression. Activation of *de novo* lipogenesis in cancer cells, which is increasingly recognized as one of the characteristics of aggressive cancers [4], correlates with a poorer prognosis and shorter disease-free survival in many tumor types including CRC [5, 6].

Abnormally elevated *de novo* lipogenesis provides cancer cells with membrane building blocks, signaling lipid molecules, and posttranslational modifications of proteins, as well as energy to support rapid cell proliferation [7]. Our published studies demonstrate an increase in expression of fatty acid synthase (FASN), a key enzyme of *de novo* lipogenesis, with advancing stages of CRC, suggesting an important role of *de novo* lipid synthesis in progression of this disease [6, 8].

The tumor-associated expression of FASN and its pro-tumorigenic functions characterized in multiple studies make this enzyme an attractive target for anti-cancer therapy [5–7, 9]. In recent years, several compounds have been reported to inhibit the enzymatic activity of FASN and reduce growth of malignant cells; however, some of them exhibit pharmacological limitations or induce weight loss, preventing their development as systemic drugs [9, 10]. In contrast, potent FASN inhibitors developed by 3-V Biosciences demonstrate anti-tumor activity *in vitro* and *in vivo* and a favorable tolerability profile in a Phase I clinical trial in patients bearing solid tumors [4, 11, 12]. The effect of FASN inhibitors on CRC progression and CRC characteristics associated with responsiveness to FASN inhibition are not yet known.

Patient-derived xenograft (PDX) models including CRC PDXs retain the intratumoral clonal heterogeneity, tumor microenvironment, and morphology of the parent tumor through passages in mice [2, 13–15] and are one of the most valuable models for preclinical drug evaluation [16]. In this study, we tested the effect of TVB-3664 and its analogs (3V-Biosciences) on tumor growth in nine CRC PDX models, primary CRC cells, and established CRC cell lines. We demonstrated that CRC PDXs and CRC cells show a wide range of sensitivity to FASN inhibition and that high levels of FASN expression are associated with increased sensitivity of cancer cells to TVB inhibitors *in vitro*. Furthermore, our results suggest that Akt, Erk1/2 and AMPK are major oncogenic pathways altered by FASN inhibitors. Finally, we showed that FASN inhibition in PDX tumor tissues is associated with a significant decrease in the pool of adenine nucleotides (AXP) which play a crucial role in a variety of metabolic functions of proliferating cells [17]. Moreover, TVB-3664 treatment significantly decreased the abundance of FA and phospholipids such as phosphatidylcholines and phosphatidic acids and significantly increased the levels of lactosylceramide and sphingomyelin in analyzed PDX models.

Together, our findings provide evidence of biological activity of TVB compounds in CRC and warrant further investigation to understand the mechanisms of resistance to FASN inhibition and improve efficacy of FASN-targeted therapy.

## RESULTS

### TVB inhibitors show anti-tumor activity in established CRC cell lines and primary CRC cells

**Figure 1:**
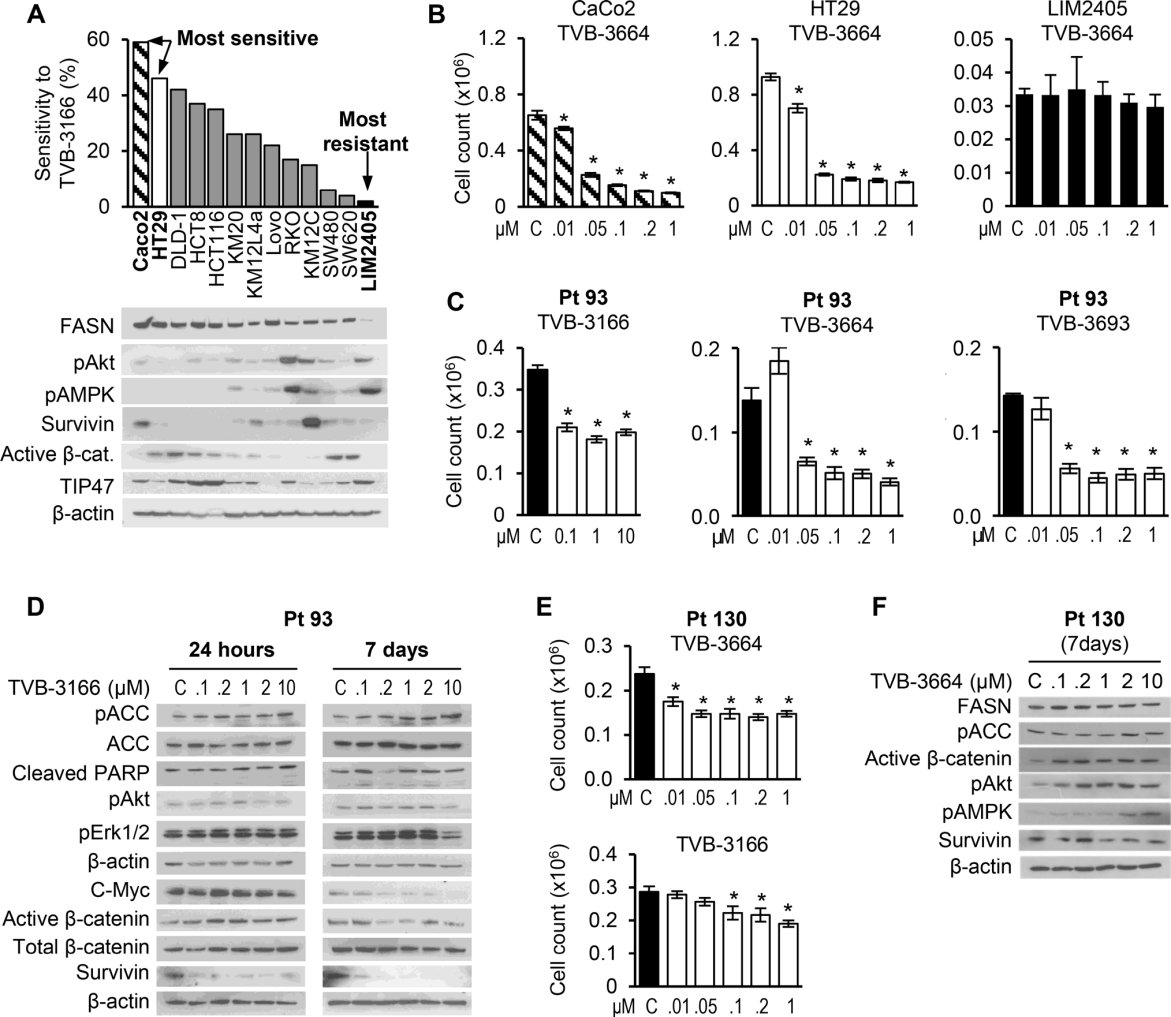
CRC cell lines exhibit a wide range of sensitivity to FASN inhibition (**A**) A panel of CRC cell lines was treated with TVB-3166 (0.2 μM) for 7 days in their normal medium supplemented with 10% FBS. Medium and drug were changed on day 4. Proliferation was determined by cell count. Data shown as percent response to FASN inhibition. (**B**) The two most sensitive (CaCo2 and HT29) and the most resistant (LIM2405) cell lines were treated with multiple concentrations of TVB-3664 for 7 days without medium change and the number of cells was counted (^*^p < 0.05). (**C**, **E**) Primary CRC cells Pt 93 and Pt 130 were treated with multiple concentrations of TVBs for 7 days and proliferation was determined by cell count. ^*^p < 0.05. (**D**, **F**) Primary CRC cells were treated for 24 h (Pt 93) and 7 days (Pt 93 and Pt 130) with TVB inhibitors and cell lyses were subjected to western blot analysis.

FASN and *de novo* lipogenesis, its cognate pathway, are critical components of tumor cell survival and proliferation for a wide range of cancers [18]. Recent studies showed that TVB-3166—an orally available, reversible, potent, and selective FASN inhibitor—decreases viability in multiple tumor cell lines from solid and hematopoietic tumor types and tumor growth of patient-derived non-small-cell lung cancer xenograft tumors [12]. To evaluate the effect of this inhibitor in CRC, a panel of 13 CRC cell lines was treated with 0.2 μM of TVB-3166 for 7 days with medium and drug change on day 4 and cellular proliferation assessed by cell count. The CRC cell lines exhibited a wide range of sensitivity to FASN inhibition, with low expression of FASN in SW480, SW620 and LIM2405 associated with resistance to FASN inhibition. Increased resistance of CRC cells to FASN inhibition appears to be associated with an increase in basal level of activation of pAkt and pAMPK. Furthermore, a subset of cell lines sensitive to TVB-3166 showed enrichment in the cell lines with a high level of active beta-catenin (Figure 1A). Similar responses were observed when CaCo2, HT29 and LIM2405 cells were treated with TVB-3664 and TVB-3693, analogs of TVB-3166, in cell line specific medium supplemented with 10% FBS for 7 days without medium change (Figure 1B; Supplementary Figure 1A). In comparison, the increase in efficacy of FASN inhibitors was due to culturing cells without a medium change, suggesting that an availability of exogenous FAs may in part compensate for the effect of these inhibitors on cellular proliferation. Consistent with our previous findings using shRNA-mediated knockdown of FASN [6], pharmacological inhibition of FASN with TVB-3166 induced apoptosis in HCT116 and HT29 cells (Supplementary Figure 1B) suggesting that TVB inhibitors have cytotoxic effect on CRC cells.

To further evaluate the effect of FASN inhibition in CRC, we treated two primary cell lines (Pt 93 and Pt 130) established from PDX tumors [19] with different doses of TVB-3166, TVB-3664 and TVB-3693. Primary CRC cells used in this study were established from PDX tumors and were used as 2D cultures (Supplementary Figure 1C–1D).

We observed a significant decrease in cellular proliferation in both cell lines (Figure 1C and 1E). However, the response to FASN inhibition was more prominent in the Pt 93 cells and associated with a significant decrease in expression of active β-catenin, c-Myc, survivin and induction of PARP cleavage (Figure 1D). No changes in pAkt and pAMPK were observed due to TVB treatment in this cell line. In contrast, FASN inhibition in Pt 130 cells lead to activation of pAMPK and pAkt pathways as well as an increase in active β-catenin (Figure 1F). These data further suggest that activation of Akt and AMPK pathways can be associated with reduced sensitivity to FASN inhibition in CRC cells.

These data demonstrate that TVB inhibitors have anti-tumor activity in CRC and suggest that the level of FASN expression and the activity of oncogenic pathways such as Akt and AMPK may determine the responsiveness of CRC to FASN inhibition.

### PDXs show a wide range of sensitivity to FASN inhibition

Expression of FASN is significantly upregulated in a stage-dependent manner in CRC [6]. To evaluate the expression of FASN in a population of patients treated at the University of Kentucky, we analyzed FASN expression in matched normal colon mucosa and tumor tissues from 56 patients with Stage I-IV CRC who had surgery at UK Chandler Medical Center. The tissue microarray results demonstrated that 9% of cases were negative for FASN expression in tumor tissues and 91% of tumors had a significant increase in FASN expression (Supplementary Figure 2A).

**Figure 2:**
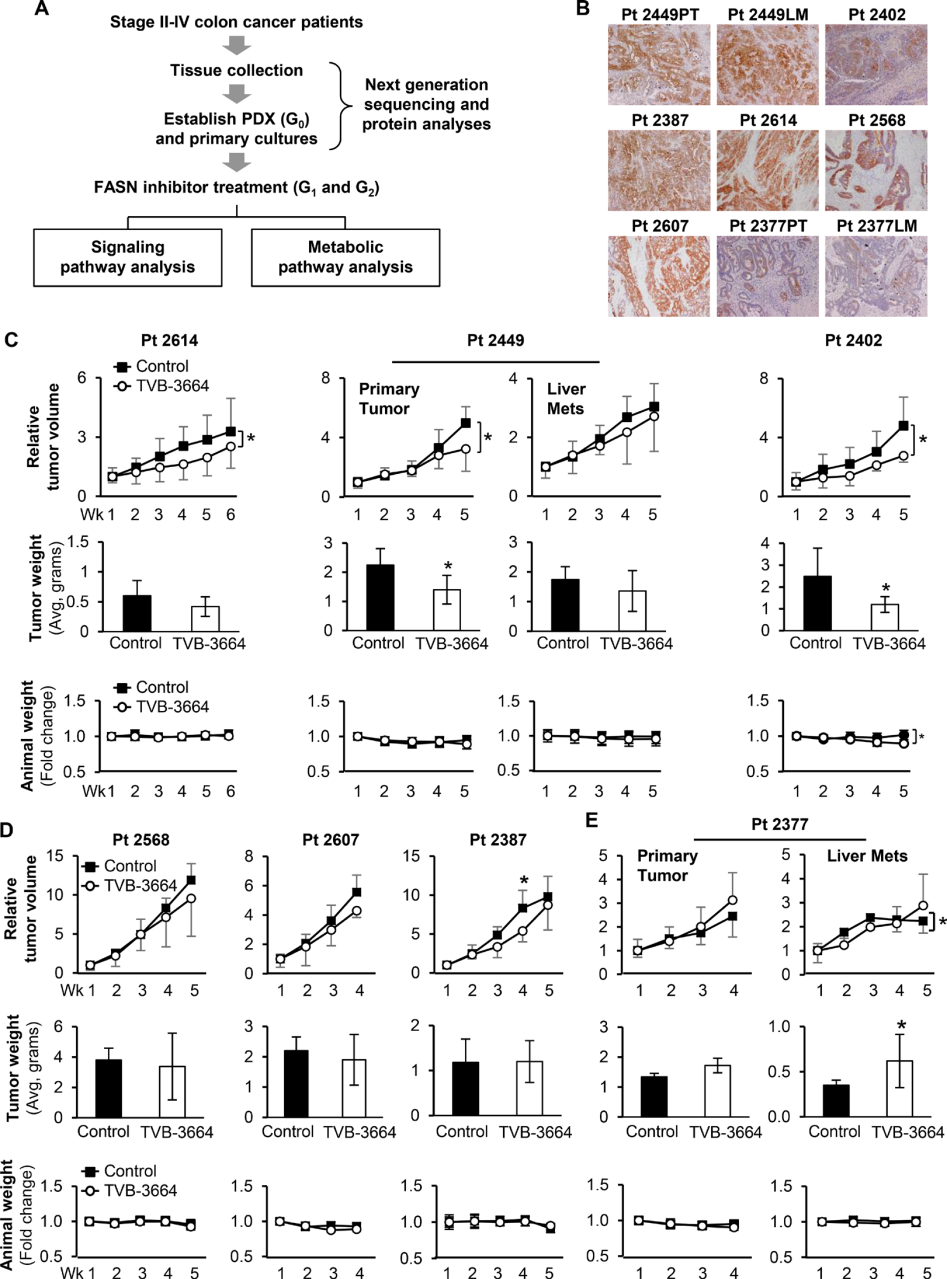
Effect of TVB-3664 on tumor growth in CRC PDX models (**A**) Schematic representation of study design. (**B**) Representative IHC images taken from resected patient tumor tissues stained for FASN expression. (**C**) PDX models sensitive to TVB-3664 treatment. Tumor response to FASN inhibition is shown as a fold change in tumor volume over time. Middle row shows corresponding tumor size at the end of the experiment. Bottom row shows corresponding fold change in weight of animals over time. Animals were treated daily with 3 mg/kg (Pt 2614 and Pt 2449PT) or 6 mg/kg (Pt 2402 and Pt 2449LM) of TVB-3664 by oral gavage (^*^p < 0.05; Pt 2377PT and Pt 2377LM are established from matched primary and liver metastasis tissues from Pt 2377). No response to TVB-3664 treatment was observed in PDX Pt 2449LM. (**D**) PDX models resistant to FASN inhibition. Tumor response to FASN inhibition is shown as a fold change in tumor volume over time. Middle row shows corresponding tumor size at the end of the experiment. Bottom row shows corresponding fold change in weight of animals over time. Animals were treated daily with 3 mg/kg of TVB-3664 by oral gavage (^*^p < 0.05). (**E**) Accelerated tumor growth in Pt 2377 PDX models treated with 3 mg/kg of TVB-3664. Tumor response to FASN inhibition is shown as a fold change in tumor volume over time in PDX models established from primary CRC (left) and liver metastasis tumors (right) Pt 2377. Middle row shows corresponding tumor size at the end of the experiment. Bottom row shows corresponding fold change in weight of animals over time (^*^p < 0.05).

TVB-3166 inhibits tumor growth of patient-derived non-small-cell lung cancer xenograft tumors [12, 20]. To evaluate the effect of TVB-3664, a newer analog of TVB-3166 [20], in CRC, we established nine CRC PDX models in NOD-SCID-IL2rg-/- (NSG) mice using specimens collected from patients who had undergone surgery for resection of primary CRC or CRC metastasis (Figure 2A). Pt 2377PT/Pt 2377LM and Pt 2449PT/Pt 2449LM models represent matching primary tumor (PT) and liver metastasis (LM) tissues collected from the same patient and established in NSG mice (Supplementary Table 1). PDX models established from patient tissues were designated generation 0 (G0); subsequent generations established through propagation of tumor tissues from mouse to mouse were designated G1 and G2. Consistent with previous reports [13, 21], the morphology of established CRC PDXs resembled the morphology of the parent tumor through passaging in mice (Supplementary Figure 2B–2C). To ensure that PDX models and established primary cells recapitulate genetic characteristics of patient tumors, the mutational status of 198 oncogenes was analyzed in patient tissues, PDX tissues and primary cells using next-generation sequencing (Table 1 and Supplementary Table 2).

**Table 1:** Mutational profile of PDX models

	APC	BRAF	KRAS	NRAS	PI3K	EGFR	FGFR	TP53
Pt 93^*^		mut	mut					
Pt 130^*^		mut	mut				mut	
Pt 2449PT	mut	mut						
Pt 2449LM		mut			mut		mut	mut
Pt 2402								mut
Pt 2614	mut			mut				mut
Pt 2607		mut				mut		
Pt 2568	mut							mut
Pt 2387	mut		mut		mut			mut
Pt 2377PT	mut		mut		mut			
Pt 2377LM	mut		mut		mut			

Mutational status of 198 cancer genes was assessed in tumor tissues obtained from CRC patients and in established PDX models and primary cells (^*^) by NSG sequencing. Mutational status of key oncogenes involved in CRC development and progression is shown (see Methods).

Immunohistological analysis of patient tumor tissues showed that all cases were positive for FASN expression, with the lowest expression observed in Pt 2377PT and LM (Figure 2B). Similar to our *in vitro* data (Figure 1), PDX models exhibited a wide range of sensitivity to FASN inhibition. TVB-3664 treatment led to a significant reduction in tumor volume and tumor weight in Pt 2614, Pt 2449PT, and Pt 2402 PDX models, with an average reduction in tumor weight of 30%, 37.5% and 51.5%, respectively (Figure 2C). Interestingly, TVB-3664 treatment of the Pt 2387 model led to a significant 35% decrease in tumor volume at week 4 followed by resistance, which developed at week 5. A small decrease in tumor volume and tumor size was observed in Pt 2449LM, Pt 2568, and Pt 2607 PDX models treated with TVB-3664, but these changes were not significant (Figure 2C, 2D). TVB-3664 treatment had an adverse effect and accelerated tumor growth in Pt 2377PT and LM PDXs (Figure 2E). Consistent with the previous report that TVB compounds are well tolerated *in vivo* [12], we did not observe any drug-related toxicity in our animal studies. With the exception of the Pt 2402 model, no significant weight loss was noted at the end of the treatment period (Figure 2C–2E).

We did not observe clear correlation between levels of FASN expression in patient tissues and the response to FASN inhibition. However, similar to *in vitro* studies (Figure 1), low expression of FASN in Pt 2377PT and LM was associated with resistance to FASN inhibition. Interestingly, Pt 2377PT and LM PDXs showed an adverse effect to long-term treatment with TVB-3664; however, these changes were not significant in Pt 2377PT. We noted that Pt 2377PT and LM models have co-existing KRAS and PI3KCA mutations (Figure 2E, Table 1). The PI3KCA mutation was also present in Pt 2387, which developed resistance during treatment (Figure 2D, Table 1). TP53 mutation was present in PDX models that were sensitive to FASN inhibition (Table 1). Even though we did not determine significant correlation between tumor mutational backgrounds and response to FASN inhibition, these findings will be tested in future studies.

Together, these findings suggest that TVB-3664 as a monotherapy has anti-tumor activity in CRC; however, studies with a larger number of cases are required to establish reliable biomarkers to identify a population of patients that is sensitive to FASN-targeted therapy.

### Activation of Akt and AMPK pathways is associated with resistance to TVB-3664 treatment in PDX models and primary CRC cells

The dependence of tumors on *de novo* lipid synthesis for their metabolic requirements, survival and growth suggests that FASN expression level may determine a tumors’ response to FASN inhibition [4, 22, 23]. Moreover, our published studies demonstrate that inhibition of FASN alters multiple signaling pathways including the Akt and AMPK pathways [6, 8].

**Figure 3:**
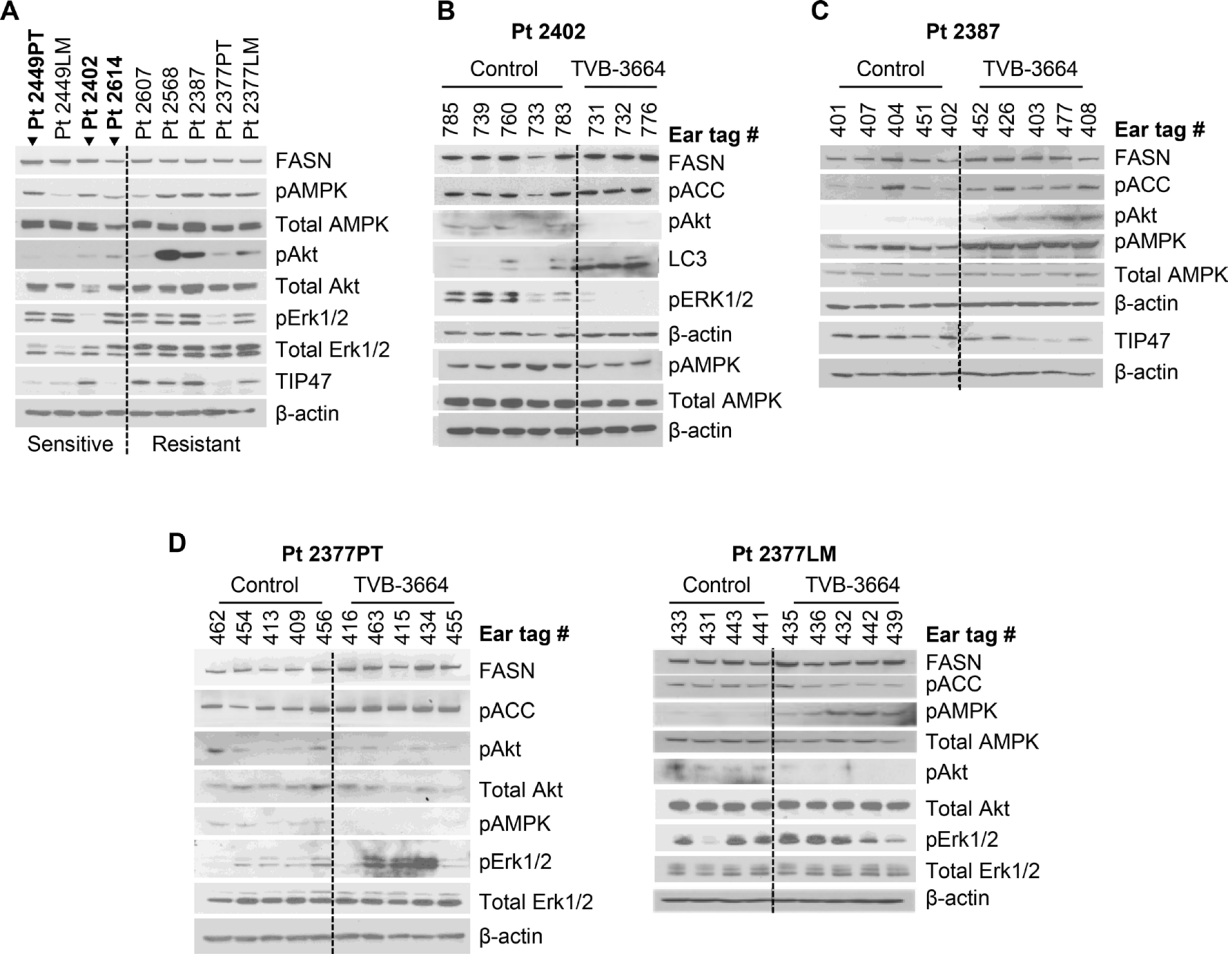
Expression of FASN and activation of FASN-associated oncogenic pathways in PDX models (**A**) Western blot analysis of tissues from PDX models (all G1; Pt 2387 G2) showing expression/activation of major oncogenic pathways associated with *de novo* lipogenesis. Cases sensitive to FASN inhibition are shown in bold (arrows). (**B**) Western blot analysis of tumor tissues from Pt 2402 treated with 6 mg/kg of TVB-3664 daily for 5 weeks. (**C**) Western blot analysis of tissues the Pt 2387 model treated with 3 mg/kg of TVB-3664 daily for 5 weeks. (**D**) Western blot analysis of tumor tissues from Pt 2377PT and LM treated with 3 mg/kg of TVB-3664 daily for 4 weeks.

To evaluate the correlation between FASN expression, activation of major oncogenic pathways, and tumor response *in vivo*, we analyzed the levels of FASN, pAkt, pAMPK, pErk1/2, and TIP47 in tumor tissues from G0 PDX models. Western blot analysis showed expression of FASN in all established PDXs, with the highest expression in Pt 2449PT and LM and in Pt 2402. We found higher levels of pAkt and pAMPK among PDX models that were resistant to FASN inhibition. The level of TIP47, a marker for lipid droplets [24], was also higher in more resistant models (Figure 3A). Pharmacological inhibition of FASN leads to a decrease in activation of pAkt [20, 25, 26]. However, inhibition of FASN also induces pro-survival Akt and ERK signaling in K-Ras-driven cancer cells [27]. To analyze the effect of TVB-3664 treatment on activity of these pathways, control and TVB-3664 treated PDX tumors were subjected to western blot analysis. The response to FASN inhibition was associated with a decrease in activation of pErk1/2 and TIP47 and induction of LC3 (an autophagy marker [28]) in the Pt 2402 model (Figure 3B). No significant changes in pAMPK or pAkt activation were observed in the other PDX models that were identified as sensitive to FASN inhibition. In contrast, TVB-3664 treatment induced activation of pAkt and pAMPK but decreased the level of TIP47 in the Pt 2387 model (Figure 3C), suggesting that activation of these pathways may be potential mechanisms of resistance to FASN inhibition. The analysis of tumor samples from TVB-3664 treated Pt 2377PT and LM models showed an increase in pErk1/2 and activation of pAMPK, respectively (Figure 3D).

All together, these data suggest that activation of AMPK, Akt and Erk1/2 pathways can be a mechanism of resistance to FASN inhibition.

### Inhibition of FASN is associated with a significant decrease in a pool of adenine nucleotides in PDX tumors

Stable knockdown of FASN decreases cellular respiration and ATP level under metabolic stress conditions in CRC cell lines [8]. Our studies using HCT116 cells, control and stable FASN knockdown, labeled with ^13^C_6_-glucose for 24 h showed that knockdown of FASN decreases lactate production by 20% and lowers the lactate to glucose ratio (by 13C-Lac /13C-Glc) 22% (*p* = 0.0026). Furthermore, FASN knockdown decreased the level of excreted lactate and acetate (*p* = 0.06) in this cell line (Supplementary Figure 3A–3D). To investigate the effect of TVB-3664 on tumor metabolism in PDX tumors, we performed untargeted metabolomics on tissue samples from Pt 2449PT, Pt 2402, and Pt 2614, which responded to FASN inhibition. Pt 2449PT and Pt 2402 mice were fasted 16 h then injected i.p. with 2 g/kg^–1^ body mass of ^13^C_6_-glucose 1 h before sacrifice, as previously described [29], which allows SIRM tracing of cellular metabolites in control and treated tumors. Mice with Pt 2614 tumors were fasted for 16 h but ^13^C_6_-glucose labeling was not performed. To prepare samples for SIRM analysis, tumor tissues were ground and polar and lipid fractions were extracted and analyzed using nuclear magnetic resonance and mass spectrometry.

**Figure 4:**
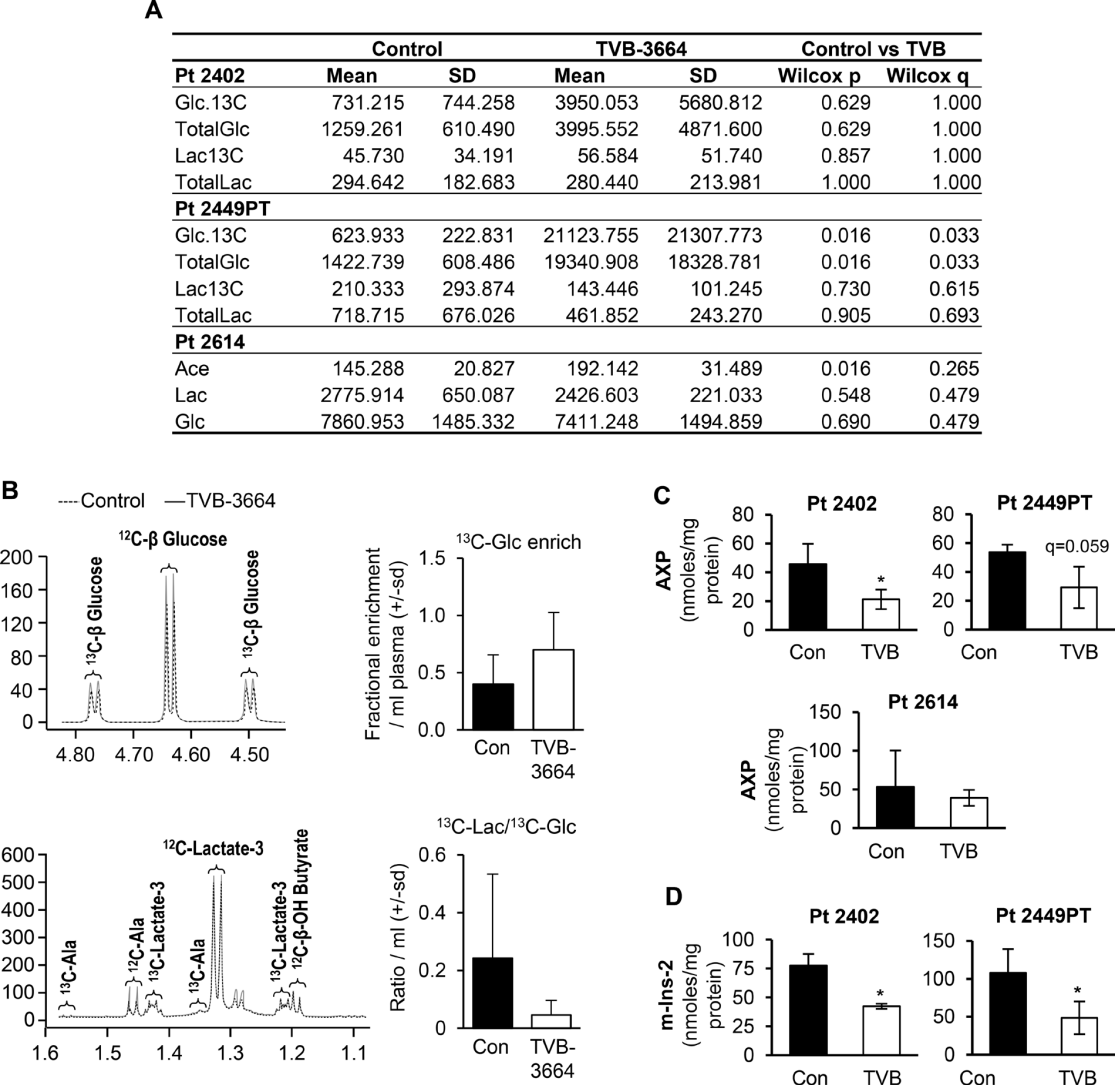
Inhibition of FASN alters metabolites levels in PDX models sensitive to TVB-3664 (**A**) Changes in plasma metabolites in Pt 2402, Pt 2449PT and Pt 2614 PDXs. (**B**) Representative figures of NMR spectra obtained from analysis of plasma from the Pt 2402 model (control vs TVB-3664 treated). Levels of AXP (**C**) and m-Ins-2 (**D**) in tumor tissues from PDX models sensitive to TVB-3664 (*p* < 0.05) (see Methods).

Analysis of plasma metabolites revealed that inhibition of FASN in Pt 2449PT and Pt 2402 led to an increase in 13C labeled glucose and total glucose in plasma samples of mice treated with TVB-3664 versus control. In Pt 2614, we observed a significant increase in the level of acetate (but not glucose) in plasma from mice treated with TVB-3664, suggesting a different metabolic response to FASN inhibition in this PDX model. In contrast to our data from FASN knockdown cells, no significant changes were observed in the levels of 13C lactate or total lactate between control and TVB-3664 treated groups (Figure 4A); however, we observed a similar decrease in the lactate to glucose ratio (13C-Lac /13C-Glc) in Pt 2402 and Pt 2449PT (Figure 4B)

The analysis of FASN-mediated changes in polar metabolites in tumor tissues showed a significant decrease in the pool of free adenine nucleotides (ATP + ADP + AM*P* = AXP) in all three PDX models analyzed; however, these changes did not reach statistical significance in the Pt 2614 model (Figure 4C). Furthermore, inhibition of FASN significantly decreased the levels of myo-Inositol (a metabolite that plays an important role in cell signaling and is a component of structural lipids [30]), in Pt 2402 and Pt 2449PT, but not Pt 2614 (Figure 4D).

Together, these data suggest that FASN inhibition is associated with an increase in glucose metabolism and a reduction in a pool of adenine nucleotides.

### Inhibition of FASN leads to significant changes in lipid composition in CRC

Stable knockdown of FASN is associated with a significant decrease in *de novo* palmitate synthesis [6] and formation of lipid droplets [8], suggesting that expression of FASN and its activity significantly affects lipid composition and storage in cancer cells. Consistently, analysis of HT29 cells (control and stable FASN knockdown) for incorporation of ^13^C_6_-glucose tracer into lipids showed that inhibition of FASN is associated with a 40% decrease in *de novo* synthesized triglycerides and phospholipids such as phosphatidylethanolamines and phosphatidylcholines, which are major components of biological membranes [31] (Supplementary Figure 4A).

**Figure 5:**
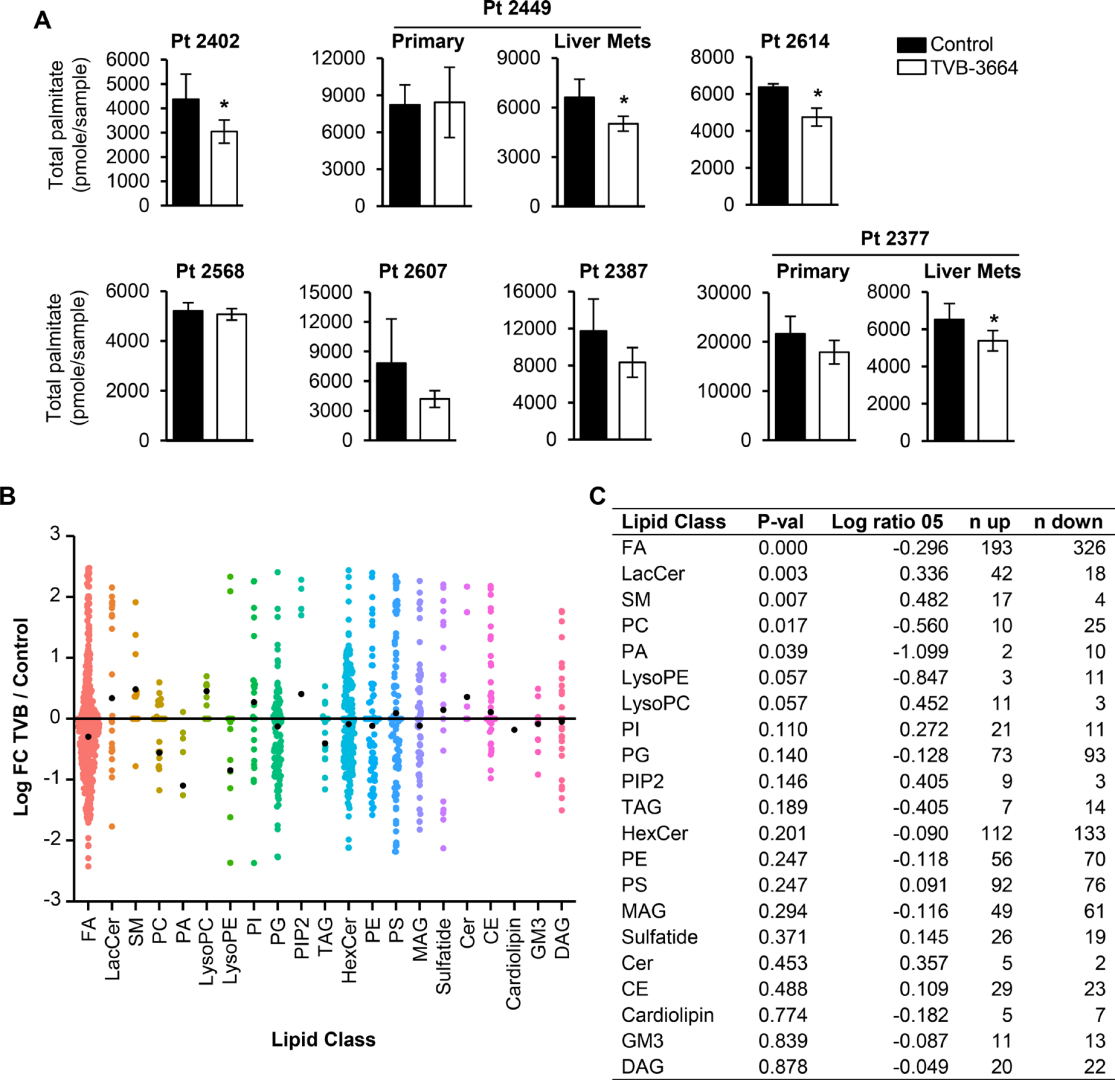
Inhibition of FASN alters lipid composition in PDX models sensitive to TVB-3664 (**A**) Total plasma palmitate levels in control and TVB-3664-treated mice measured by mass spectrometry. FA (50 μl of a 1 μM C17) was added to 50 μl of plasma as an internal standard. Half of each sample was saponificated and derivatized and total palmitate measured by mass spectrometry (^*^p < 0.05). (**B**–**C**) FASN-mediated changes in lipid classes common among Pt 2402, Pt 2449PT and Pt 2614 PDX models. Lipid classes were evaluated by grouping the lipids to a class, and within each class setting lipids with fold-change >0 as successes, and <0 as failures, and testing the ratio of successes to failures to 0.5 using a two-sided binomial test. The reported value for the binomial test is the log-ratio of the calculated proportion of successes over 0.5 (see Methods).

To assess the effect of TVB-3664 on lipid metabolism in PDX models, we first analyzed the levels of palmitate in plasma samples collected from control and TVB-3664 treated mice prior to their sacrifice. Plasma samples were analyzed by mass spectroscopy as previously described [6]. We observed a significant decrease in total palmitate levels in Pt 2402, Pt 2449LM, Pt 2614 and Pt 2377LM but not in others (Figure 5A), suggesting that there is no correlation between pharmacological inhibition of FASN and the level of total palmitate in plasma. Furthermore, we analyzed the lipid fractions extracted from control and TVB-3664 treated tumors for Pt 2449PT, Pt 2402 and Pt 2614 models. TVB-3664 treatment led to a distinct lipid profile in each model as shown by principal component analysis (Supplementary Figure 4B–4D). The table of lipid species that significantly changed in each PDX model is shown in Supplementary Figure 4E. The binominal analysis of common changes in lipids among these three PDX models showed that FASN inhibition is associated with a significant decrease in the levels of fatty acids and phospholipids such as phosphatidylcholines and phosphatidic acids and a significant increase in levels of lactosylceramide and sphingomyelin (Figure 5B, 5C).

In summary, these data further suggest that anti-cancer effect of FASN inhibition is associated with a significant impact on lipid composition.

## DISCUSSION

An increase in *de novo* lipid synthesis is the common characteristic of many tumors including colorectal cancer [5]. Inhibition of FASN decreases proliferation and diminishes metastatic capabilities of cancer cells in multiple tumor models [4, 6, 18, 32]. Currently, novel FASN inhibitors developed by 3V-Biosciences are being evaluated in Phase I/II clinical trial [33]. TVB-2640 demonstrates prolonged stable disease when given in monotherapy and partial response when combined with paclitaxel in KRASmut non-small cell lung, ovarian, and breast cancer patients [18]. However, the effect of FASN inhibition in CRC and potential mechanisms of resistance to FASN inhibition are still not fully understood. This study is the first to evaluate the effect of TVB inhibitors in CRC using PDX models and primary CRC cells.

Evaluation of FASN expression in a CRC tissue microarray showed that 91% of patients who have undergone surgery at the University of Kentucky are positive for FASN expression, which is consistent with previously published data [34]. All nine PDX models used for evaluation of TVB-3664 in the current study were positive for FASN expression. Treatment of CRC PDXs with TVB-3664 as a monotherapy led to a significant decrease in tumor volume in 30% of cases suggesting that the presence of FASN expression does not predict response to FASN inhibitors. The maximum reduction in tumor weight in response to TVB-3664 treatment achieved in our study was 50% which is significantly less than the >80% reduction in tumor growth in non-small-cell lung cancer PDXs treated with TVB-3166 [12] suggesting that lung cancer may be more susceptible to FASN inhibition.

Consistent with the study showing variability in sensitivity to TVB-3166 among 90 cell lines from different tumor types [12], our *in vitro* studies demonstrated a significant variability in responses to FASN inhibition in CRC. In contrast to reported data from other tumor types [12, 35], we noted a correlation between high expression of FASN and increased sensitivity to FASN inhibition in established CRC cell lines. However, we did not identify a similar correlation in *in vivo* studies, suggesting that other factors such diet and/or the ability of particular tumors to metabolize endogenous fatty acids may contribute to the tumor’s response to TVBs as well. Similar to LIM2405, which was not sensitive to FASN inhibition due to very low expression of the enzyme in this cell line, low levels of FASN expression in Pt 2377PT and LM PDXs were associated with resistance to TVB-3664 and prolonged treatment led to acceleration of tumor growth. All together, *in vitro* and *in vivo* data suggest that evaluation of FASN expression is important for selection of candidates to FASN-targeted therapy, but the tumor being positive for FASN expression is not sufficient to predict response to this therapy.

In attempt to correlate the response of FASN inhibition to the mutational profile of tumors, we assessed the mutational status of 198 cancer-related genes. Studies performed by 3V- Biosciences [12] have shown an association of FASN inhibitor sensitivity with KRAS mutation status in lung cancer cell lines, but not in analysis of 29 CRC cell lines. Consistently, we did not identify any correlation between presence of KRAS mutation and response to FASN inhibition. Furthermore, PI3K/Akt signaling has been linked to FASN expression in many tumor types [26, 27, 36, 37]. Despite the low number of cases analyzed in this study, we noted enrichment of mutant PI3K in PDXs resistant to FASN inhibition. In contrast to the previously published study which showed no correlation between PI3K status and CRC cell lines sensitive to FASN inhibition [12], our data from analysis of cell lines and PDX models treated with TVB inhibitors suggest that PI3K mutational status and activation of Akt downstream may affect the response of CRC cells to FASN inhibition. Our findings are supported by other reports showing that activation of the PI3K-Akt axis in response to FASN inhibition may be a potential mechanism of resistance to FASN inhibition [26, 27]. The role of PI3K/Akt pathway in resistance to FASN inhibition is currently being investigated in our laboratory.

Dysregulation of p53 tumor suppressor gene is one of the most frequent events contributing to transformation of normal cells to CRC and is associated with the aggressive and metastatic features of this disease [36]. We noted the presence of a mutation in TP53 genes in all PDX models sensitive to FASN inhibition suggesting a potential link between the TP53 status and the response to TVB-3664.

To investigate other possible markers of FASN sensitivity, the effect of TVB-3664 on the composition of metabolites and lipids was analyzed in 3 PDX models sensitive to FASN inhibition. In agreement with our reports and others [8, 18, 35], FASN inhibition led to a decrease in the levels of fatty acids, phospholipids, and triacylglycerols. Our finding that a high level of TIP47 is associated with resistance to TVB-3664 suggests that tumors with higher levels of stored lipids are more resistant to FASN inhibition.

The tumor-suppressing signaling lipid ceramide is elevated upon FASN inhibition in 231MFP cells [12] and this is consistent with our data showing increased levels of ceramide and hexaceramide in Pt 2402 and Pt 2449PT, respectively. Our results also show an increase in levels of lactosylceramide and sphingomyelin in TVB-3664 sensitive tumors, which has not been previously reported. Sphingolipids are a class of bioactive lipids that are key modulators of multiple pathophysiologic processes including tumorigenesis. Our ongoing studies investigate the role of FASN-mediated sphingolipid metabolism in CRC progression and metastasis.

Analysis of metabolites, showing a decrease in a pool of AXPs and Inositol in TVB-3664 treated PDX tumors, supports our previously published data that FASN promotes survival of CRC cells via upregulation of cellular respiration [8].

Combination of FASN inhibitors with other therapeutic agents can make cells sensitive to other drugs or can lead to synergetic effect [18]. Findings in this study provide evidence of activation of signaling pathways such as AMPK and Akt in response to FASN inhibition.

Therefore, combination of FASN inhibitors with inhibitors of Akt or AMPK pathways may be a potential therapeutic strategy for CRC. Moreover, our studies suggest that inhibition of *de novo* fatty acid synthesis should be combined with dietary changes or with other therapeutic agents to prevent compensatory mechanisms by exogenous lipids. A better understanding of the relative contribution of endogenous and exogenous lipids to malignant transformation and cancer progression would further advance the development of new therapeutic approaches to target lipid metabolism in CRC.

## MATERIALS AND METHODS

### Colon cancer primary and established cell lines

Primary colon cancer Pt 93, Pt 130 and Pt 2387 cultures were isolated and established from PDX tumors as previously described [38]. Cells were maintained as monolayer culture in DMEM supplemented with 10% FBS (Sigma-Aldrich, St. Louis, MO) and 1% penicillin–streptomycin.

CRC cell lines were authenticated using STR DNA profiling in March 2016 (Genetica, Cincinnati, OH). Primary Pt 93 and Pt 130 cells were authenticated as unique human cell lines (Genetica). The mutation profiles of these cell lines were determined using targeted (a panel of 198 genes) NGS (UKC Genomics_OGSRF). TVB-3664, TVB-3166 and TVB-5693 inhibitors were provided by 3V-Biosciences (Menlo Park, CA). The treatment regimen for *in vitro* studies is based on previously published studies [12, 20].

### TMA analysis

Immunoreactivity score of FASN expression was analyzed in matched normal colon mucosa and tumor tissues from patients diagnosed with Stage I-IV CRC who had surgery at UK Chandler Medical Center (TMA ID BH15991A, *n* = 57 normal and 56 tumor tissues). Scoring was carried out blindly by a pathologist. Immunoreactivity score was determined by multiplication of the values for staining intensity (0, no staining; 1, weak; 2, moderate; 3, strong staining) and the values for percentage of positive tumor cells (0, no positive cells; 1, 0–10%; 2, 11–50%; 3, 51–100% positive).

### Tissue collection and *in vivo* studies

Tissues were obtained from consented patients with Stage II-IV CRC who had undergone surgery at UK Medical Center (IRB # 16-0439-P2H). 6–8-week-old NSG mice (NOD.Cg-*Prkdc Il2rg* /SzJ) from The Jackson Laboratory (Bar Harbor, ME, USA) were used for PDX models. All procedures were performed using protocols approved by the UK Animal Care and Use Committee. Briefly, CRC tissues (2–5 mm) obtained from CRC patients of both sexes were implanted subcutaneously into their flanks in a small pocket surgically created under the skin. Established tumors were designated as generation 0 (G0). Tumor tissues from G0 were minced and mixed with Matrigel to ensure homogeneous distribution of tissues among mice and allow implantation of an equal volume of tumor tissues into the flank. To preserve histopathological and genomic characteristics of clinical CRC tumors and recapitulate the differential responses of CRC tumors to FASN inhibitors, all established PDX models were treated at G1 with exception of the Pt 2387 case, which was treated at G2. When tumors reached 100 mm3, the mice were randomized according to animal weight and tumor size. For evaluation as monotherapy, treatment (*n* = 5) and control (*n* = 5) groups were given TVB-3664 (3–6 µg/kg) vs vehicle (30% PEG400) by oral gavage daily for 4–6 weeks. Tumors were measured once per week by a digital caliper and tumor volume was calculated using the formula: TV = width^2^ × length × 0.52 as previously described [12]. Tumor weight was measured at the end of the experiment. Blood samples were collected through cardiac puncture at the time of sacrifice.

Mice were fasted and then injected *i.p.* with 2 g kg^–1^ body mass of ^13^C_6_-glucose 16 h prior to sacrifice as previously described [29], to allow SIRM tracing of cellular metabolites of tumors. 1 h after injection, blood samples were collected and separated into plasma and blood cells by centrifugation (4° C, 3,500 × g, 15 min). Mice were euthanized and tumor tissue was collected for IHC and flash frozen immediately in liquid N2 for protein and metabolic analysis.

### *In vivo* data analysis

PDX treatment data are shown as the mean and SD for the vehicle and TVB groups and statistical significance determined by a two-sided, two-sample *t*-test with 5% significance. For combination treatment, statistical significance was determined based on a one-way ANOVA with 5% significance. Analysis of tumor volume entailed a comparison at the last follow-up time using ANOVA in addition to linear mixed models for analysis of tumor growth over time and calculation of an adjusted aAUC and associated confidence interval to quantify tumor growth inhibition between each treated versus vehicle group. We employed a linear mixed model with a random effects term for the PDX model and fixed effects term for treatment group to perform an overall comparison of tumor volume across treatment groups and PDX models.

### Tissue extractions for metabolic studies

Frozen tissues were ground in liquid N_2_ to <10 µm particles in a 6750 Freezer/Mill (Retsch, Inc., Newtown, PA) and extracted simultaneously for soluble and lipidic metabolites as previously described [39].

### Nuclear magnetic resonance analysis

NMR spectra were obtained on an Agilent DD2 14.1 T spectrometer equipped with a 3-mm inverse triple resonance HCN cold probe. The 1D proton spectra were recorded at 288 k with an acquisition time of 2 s and a 4 s relaxation delay, during which the residual HOD resonance was irradiated. 1D ^1^H[^13^C]HSQC spectra were recorded with an acquisition time of 0.25 s and a 1.75 s relaxation delay.

Internal DSS-d6 was used as a chemical shift reference and for absolute quantification. MNOVA (Mrestrelab Research, Santiago de Compostela, Spain) was used for NMR spectral processing. After phasing and baseline correction, metabolites were quantified using the peak fitting routines in MNOVA, and assigned using in-house databases [40]. Isotopomers were quantified as absolute and fractional enrichments as previously described [41–43]. Assigned metabolites were normalized to mg protein determined by BCA analysis.

### Fourier transform mass spectrometry (FT-MS) analysis

A solution of chloroform/methanol (2:1, v/v) containing 1 mM butylated hydroxytoluene was used to dissolve the lipid extracts. MS analysis was performed using an Orbitrap Fusion Tribrid (Thermo Fisher Scientific, San Jose, CA, USA) attached to a TriVersa NanoMate (Advion Biosciences, Ithaca, NY, USA) using a “D” electrospray chip (nozzle inner diameter 4.1 μm) and a cooling unit to keep the samples at 4° C. For negative and positive ion mode analysis aliquots of the dissolved lipid extracts were diluted with methanol and ammonium formate in 2-propanol/methanol/chloroform. Nanoelectrospray ionization used 1.6 kV with a 0.6 psi head pressure in positive ion mode, and 1.5 kV and a 0.5 psi head pressure in the negative mode. All data were recorded in profile mode using a 100 ms maximum injection time, automated gain control at 2.5 × 10^5^, five microscans, and a target resolution of 450,000 (FWHM at m/z 200). The manufacturer’s default standard recommendations for the Orbitrap Fusion Tribrid tuning and calibration was used to achieve a mass accuracy of 0.5 ppm or less. A previously described software package developed in-house, Precalculated Exact Mass Isotopologue Search Engine (PREMISE), was used to make assignments and the isotopologue distributions for the various lipid classes [44]. The data were further processed to remove naturally occurring ^13^C enrichment in lipid species using the protocol developed by Moseley [45].

### Metabolic data analysis

Polar fraction analysis: The Wilcoxon rank-sum test was used to compare the abundance of each metabolite between TVB and control groups. The method proposed by Storey *et al.* [46] was used to adjust for multiple comparisons and calculate the false discovery rate *q*-value. Data analysis was performed using R (version 3.3.1). Metabolites with *q*-value < 0.05 were considered statistically significant.

### Lipid analysis

Assigned positive and negative lipid intensities were filtered based on HPD sites calculated from the raw peak lists for each sample. Lipid intensities were normalized by total intensities in each sample, and log-transformed after adding a small positive value that was 10e-3 smaller than the smallest intensity observed. Lipids present in 2/3 of either the control or TVB treated samples were kept. For each patient, the average log-intensity in the control samples was subtracted from the control and TVB samples, resulting in a log fold-change of TVB to control for a given patient sample.

Individual lipids were evaluated by two-sided *t*-test on the TVB samples compared to zero. Lipid classes were evaluated by grouping the lipids to a class, and within each class setting lipids with fold-change >0 as successes, and <0 as failures, and testing the ratio of successes to failures to 0.5 using a two-sided binomial test. The reported value for the binomial test is the log-ratio of the calculated proportion of successes over 0.5. All data processing was carried out in R 3.3.2.

### Next generation sequencing

NGS was performed on DNA from patient tissues and matched PDXs and primary cells. Briefly, library preparation was performed using the Agilent Bravo NGS workstation and Agilent SureSelect XT Library Prep Kit according to the manufacturer’s instructions (Agilent Technologies Inc., Santa Clara, CA). Extracted genomic DNA was quantified using the Qubit dsDNA HS Assay (Life Technologies, Illkirch, France). DNA quality was assessed using the Agilent 2200 TapeStation Genomic Screentape and reagents (Agilent Technologies Inc., Santa Clara, CA). Genomic DNA samples (500 ng) were sheared to fragment sizes of 170–200 base pairs using a Covaris E220 instrument (Covaris, Woburn, MA). Samples were adaptor-ligated and amplified for 10 cycles using a GeneAmp 9700 thermocycler (Applied Biosystems, Foster City, CA). The DNA was then purified using 1.8× volume AMPure XP beads (Beckman Coulter, Brea, CA). 750 ng of each amplified library was hybridized with biotinylated RNA baits comprising a custom panel of 198 clinically relevant oncology-related genes (Solid Tumor NGS Panel v1 Genomics Core Laboratory, UK, Lexington, KY). The hybridization was run overnight at 65° C and was followed by DNA capture with streptavidin beads. Captured libraries were amplified for 12 cycles with indexing primers and cleaned with 1.8× volume AMPure XP beads. Libraries are pooled at 4 nM and loaded at 9 pM onto the HiSeq 2500 as per manufacturer’s instructions for paired-end sequencing, with a run length of 100 bp.

## SUPPLEMENTARY MATERIALS FIGURES AND TABLES



## Abbreviations

CRC: Colorectal Cancer
FASN: Fatty Acid Synthase
LM: Liver Metastasis
NSG: NOD-SCID-IL2rg
PDX: patient-derived xenograft
PT: Primary Tumor
