# Supplementary material for: Preclinical evaluation of novel fatty acid synthase inhibitors in primary colorectal cancer cells and a patient-derived xenograft model of colorectal cancer
Source: Oncotarget. 2018 May 15;9(37):24787–800. doi: 10.18632/oncotarget.25361 (PMC5973868; doi:10.18632/oncotarget.25361)
Supplement: Supplementary file 1 [file oncotarget-09-24787-s001.pdf]

Preclinical evaluation of novel fatty acid synthase inhibitors in primary colorectal cancer cells and a patient-derived xenograft model of colorectal cancer

SUPPLEMENTARY MATERIALS

Supplementary Table 1: PDX models used for TVB-3664 evaluation

Human specimen ID	Primary staging	Age/ Gender	Pathology	PDX generation(s) used for treatment
Pt 93*	T3N1bM1	63/M	Metastatic medullary cancer, morphologically consistent with metastasis from colon (peritoneum and abdominal wall)	Cells (G3)
Pt 130*	T3N0M1a	76/M	Metastatic colonic adenocarcinoma	Cells (G3)
Pt 2377	T3N0M1a	66/F	Metastatic colonic adenocarcinoma (primary tumor and liver metastases)	PTG1/LMG1
Pt 2387	T3N0	49/F	Metastatic adenocarcinoma (lung) consistent with colorectal primary tumor	G2
Pt 2402	T3N1M1a	47/F	Metastatic adenocarcinoma (lung) consistent with colon primary tumor	G1
Pt 2449	T3N2M1a	61/F	Metastatic medullary colonic carcinoma (primary tumor and liver metastases)	PTG1/LMG1
Pt 2568	T2N0	69/F	Moderately differentiated mucinous colonic adenocarcinoma	G1
Pt 2607	T4aN1a	65/M	Mucinous colonic adenocarcinoma	G1
Pt 2614	T2N0	72/M	Moderately differentiated colonic adenocarcinoma	G1

Demographic and clinical information on CRC patients whose tissues were used to establish PDXs and primary cell lines. PDX generation used for treatment or establishing cell lines is shown.

Supplementary Table 2: Major CRC-associated mutations identified in cases used in the study

VEP_RefSeq_anno	VEP_RefSeq_effect	Gene	Gene_id	HGVS_RefSeq_cdna_change	HGVS_RefSeq_Prot_change
Pt 93					
Missense_variant	MODERATE	BRAF	673	NM_004333.4:c.1799T>A	NP_004324.2:p.Val600Glu
Splice_region_variant&synony	LOW	KRAS	3845	NM_033360.2:c.1147>C	
Pt 130					
Missense_variant	MODERATE	BRAF	673	NM_004333.4:c.1799T>A	NP_004324.2:p.Val600Glu
Missense_variant	MODERATE	FGFR1	2260	NM_001174067.1:c.2557C>T	NP_001167538.1:p.Arg853Cys
Splice_region_variant&intron_	LOW	KRAS	3845	NM_033360.2:c.-11-5A>G	-
Pt 2449					
Missense_variant	MODERATE	BRAF	673	NM_004333.4:c.1799T>A	NP_004324.2:p.Val600Glu
Missense_variant	MODERATE	TP53	7157	NM_001126112.2:c.472C>T	NP_001119584.1:p.Arg158Cys
Pt 2402					
Missense_variant	MODERATE	TP53	7157	NM_000546.5:c.818G>A	NP_000537.3p.Arg273His
Pt 2614					
Stop_gained	HIGH	APC	324	NM_001127510.2:c.4348C>T	NP_001120982.1:p.Arg1450Ter
Missense_variant	MODERATE	NRAS	4893	NM_002524.4:c.37G>C	NP_002515.1:p.Gly13Arg
Missense_variant	MODERATE	TP53	7157	NM_000546.5:c.524G>A	NP_000537.3:p.Arg175His
Pt 2607					
Missense_variant	MODERATE	EGFR	1956	NM_005228.3:c.2326C>T	NP_005219.2:p.Arg776Cys
Missense_variant	MODERATE	BRAF	673	NM_004333.4:c.1799T>A	NP_004324.2:p.Val600Glu
Pt 2368					
Stop_gained	HIGH	APC	324	NM_001127510.2:c.4161T>A	NP_001120982.1:p.Cys1387Ter
Missense_variant	MODERATE	TP53	7157	NM_000546.5:c.584T>A	NP_000537.3:p.Ile195Asn
Pt 2387					
Missense_variant	MODERATE	PIK3C2B	5287	NM_002646.3:c23G>A	NP_002637.3:p.Gly8Glu
Pt 2377					
Stop_gained	HIGH	APC	324	NM_001127510.2:c.2212A>T	NP_001120982.1:p.Lys738Ter
Frameshift_variant	HIGH	APC	324	NM_001127510.2:c.4666dup/	NP_001120982.1:p. Thr1556AsnfsTer3
Missense_variant	MODERATE	PIK3A	5290	NM_006218.2:c.1633G>A	NP_006209.2:p.Glu545Lys
Missense_variant	MODERATE	KRAS	3845	NM_033360.2:c.436G>A	NP_203524.1:p.Ala146Thr

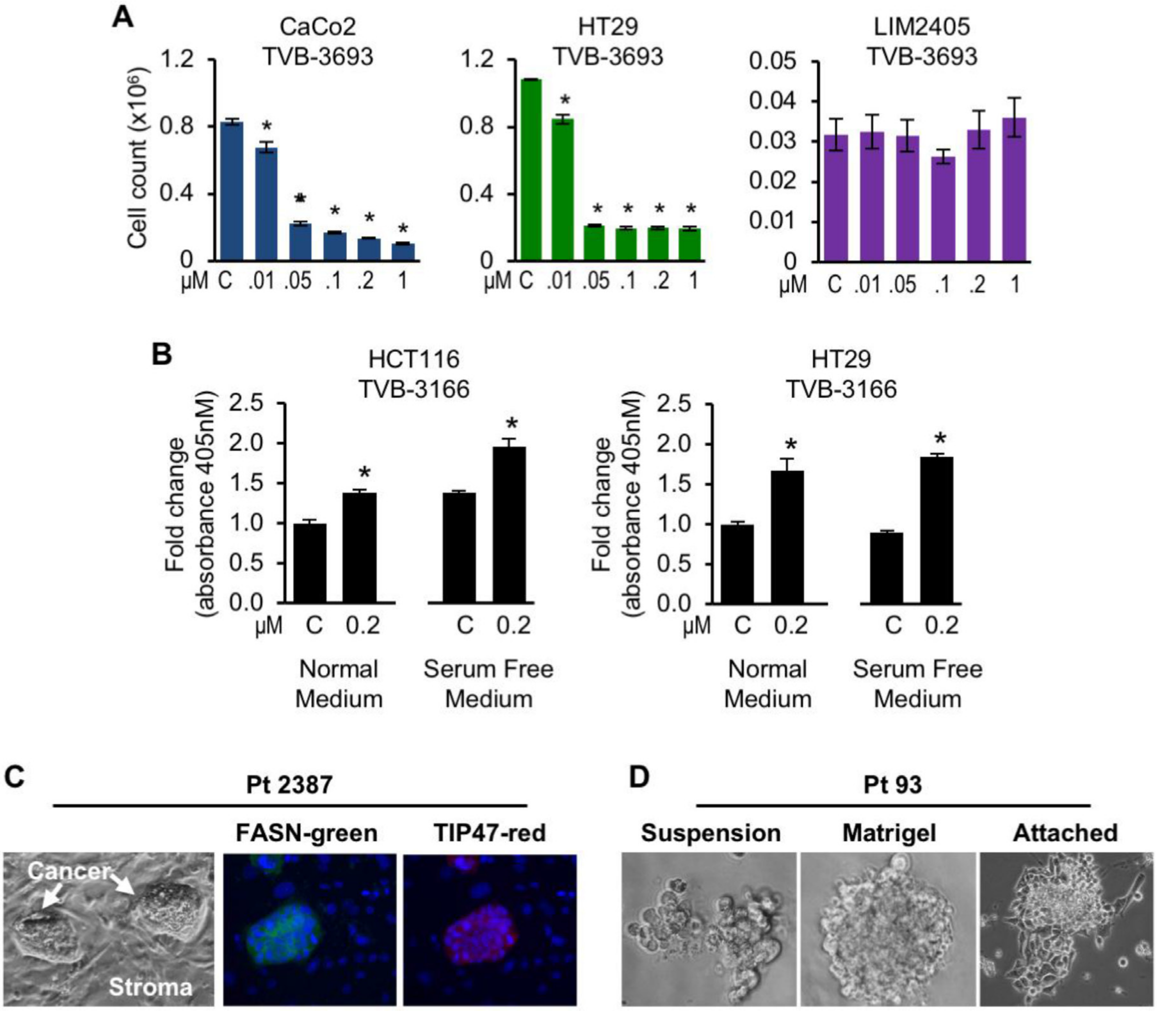

Supplementary Figure 1: (**A**) The two most sensitive (CaCo2 and HT29) and the most resistant (LIM2405) cell lines were treated with multiple concentrations of TVB-3693 for 7 days without medium change and the number of cells was counted (* $p < 0.05$). (**B**) HCT116 and HT29 cells were treated with TVB-3166 for 7 days and apoptosis was measured by Cell Death ELISA (* $p < 0.05$). (**C**) BF image and expression of FASN and TIP47 in primary culture (cancer and surrounding stromal cells) established from 1st generation PDX from Pt 2387. (**D**) Tumor organoids isolated from the primary tumor of Pt 93 grown in suspension, Matrigel and attached culture conditions.

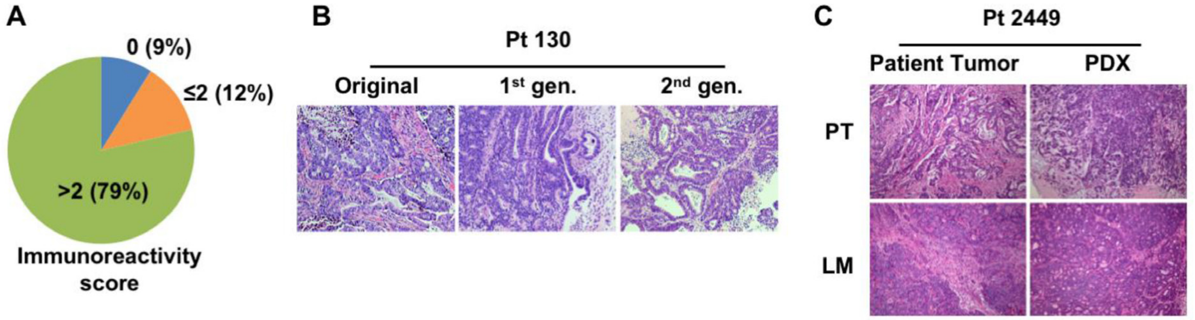

Supplementary Figure 2: FASN expression is significantly upregulated in colon cancer tissues. (**A**) Distribution of FASN immunoreactivity score was analyzed in tumor tissues from patients who were diagnosed with Stage I-IV CRC and had surgery at UK Chandler Medical Center (56 tumor tissues). (B–E) Characterization of colon cancer PDX models and primary cultures. (**B**) H&E staining of patient tumor and 1st and 2nd generation PDX tumors of Pt130. (**C**) H&E staining of patient tissues and 1st generation PDX models for Pt2449 (PT-primary tumor; LM-liver metastasis).

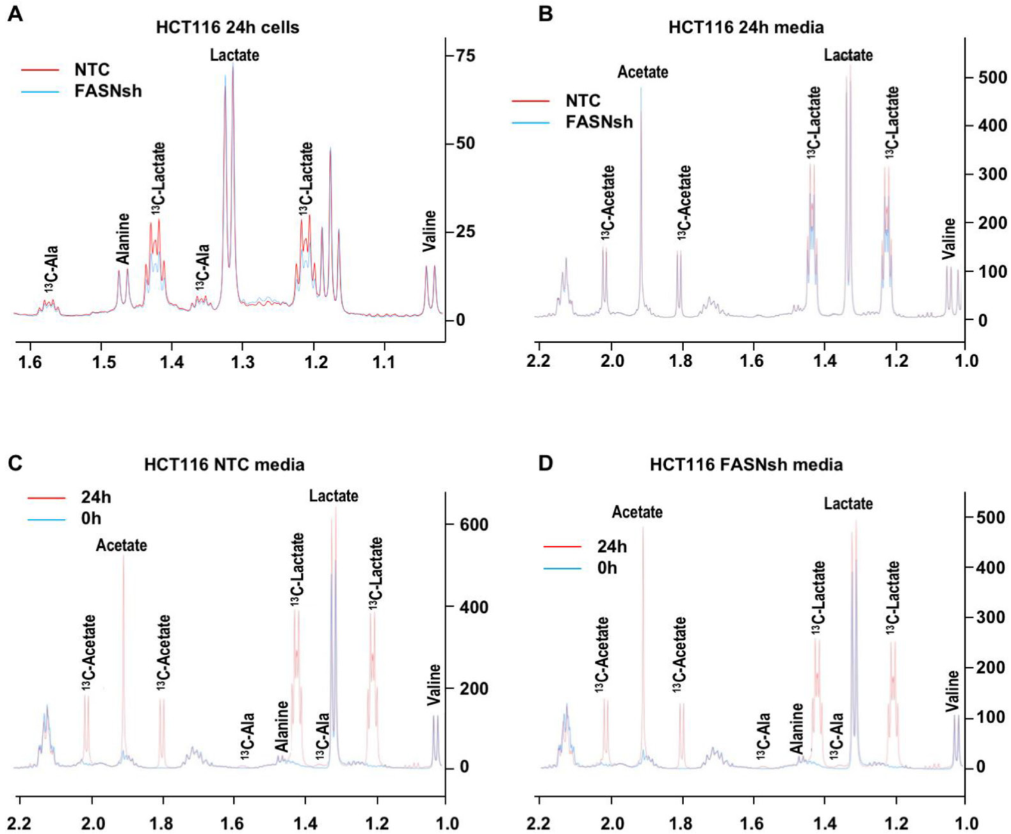

Supplementary Figure 3: Metabolite analysis in HCT116 cells. NMR spectrum of control and FASNsh cells at 24 h (**A**) and medium at 24 h (**B**)**.** NMR spectrum of medium at 0 h and 24 h of NTC (**C**) and FASNsh (**D**) cells.

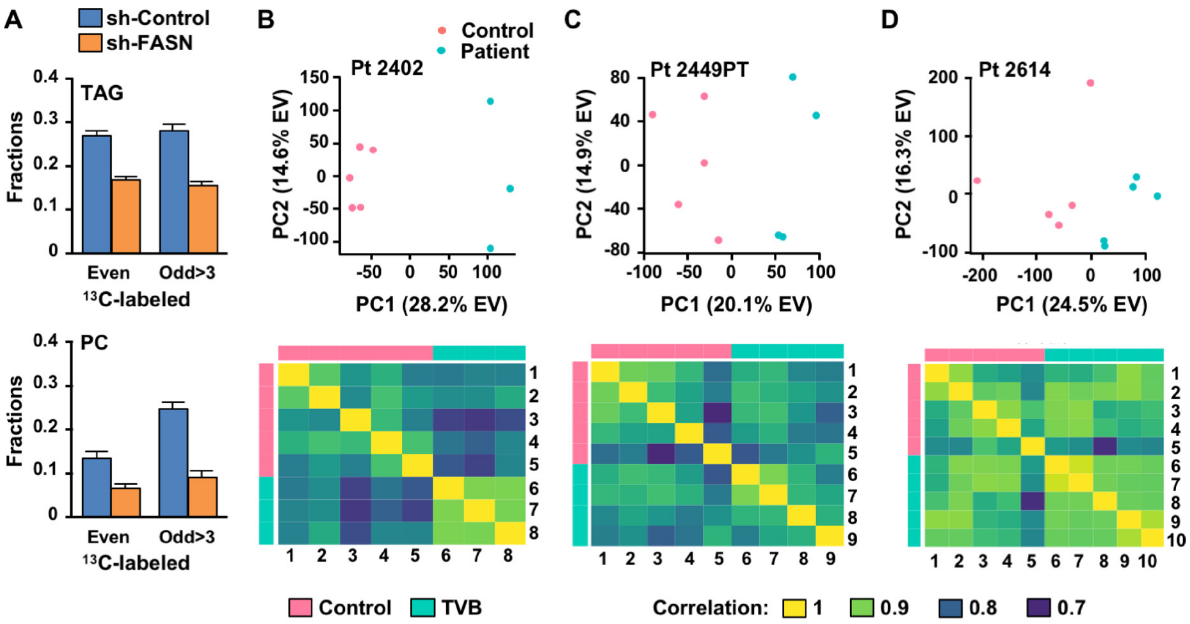

E

Pt	Diff	Control	TVN	p	Adj p	Feature	Lipid class
**Pt 2402**	17.854	-9.958	-27.812	0.000	0.000	PE.pmg.38.5.43…Na.C43H76N1O7P1..pos	PE
	19.501	-8.311	-27.812	0.000	0.000	PC.32.1.40…Na.C40H78N1O8P1..pos	PC
	20.019	-7.793	-27.812	0.000	0.000	PE.38.3.43…H.C43H80N1O8P1..pos	PE
	21.498	-6.314	-27.812	0.000	0.000	PC.38.5.46…H.C46H82N1O8P1..pos	PC
	17.825	-9.987	-27.812	0.000	0.000	TAG.57.1.60…Na.C60H114O6..pos	TAG
	15.643	-12.168	-27.812	0.000	0.000	PE.pmg.40.0.45…NH4.C45H90N1O7P1..pos	PE
	15.922	-11.890	-27.812	0.000	0.000	PS.pmg.38.3.44..HC44H82N1O9P1.0.neg	PS
	16.411	-11.401	-27.812	0.000	0.000	PS.pmg.40.5.46..HC46H82N1O9P1.0.neg	PS
	16.435	-11.377	-27.812	0.000	0.000	PS.pmg.40.4.46..HC46H84N1O9P1.0.neg	PS
	15.007	-12.805	-27.812	0.000	0.000	PS.pmg.42.4.48..HC48H88N1O9P1.0.neg	PS
	15.573	-12.239	-27.812	0.000	0.000	PS.pmg.42.5.48..HC48H86N1O9P1.0.neg	PS
	-19.271	-27.812	-8.541	0.000	0.001	PC.34.4.42…H.C42H76N1O8P1..pos	PC
	-23.174	-27.812	-4.637	0.000	0.001	TAG.52.2.55…NH4.C55H102O6..pos	TAG
	14.106	-13.706	-27.812	0.000	0.001	PE.42.9.47..M.Na.2H..C47H76N1O8P1.0.neg	PE
	-15.171	-27.812	-12.641	0.000	0.002	DAG.42.7.45…NH4.C45H74O5..pos	DAG
	-14.299	-27.812	-13.513	0.000	0.002	CE.26.4.53…NH4.C53H88O2..pos	CE
	-13.965	-27.812	-13.847	0.000	0.002	CE.14.1.41…NH4.C41H70O2..pos	CE
	-14.052	-27.812	-13.760	0.000	0.003	PE.26.6.31…H.C31H50N1O8P1..pos	PE
	-16.606	-27.812	-11.205	0.000	0.005	TAG.44.7.47…NH4.C47H76O6..pos	TAG
	-2.080	-11.675	-9.595	0.000	0.005	PS.pmg.40.4.46…H.C46H84N1O9P1..pos	PS
	-14.474	-27.812	-13.338	0.000	0.008	Cer.42.4.42…H.C42H77N1O3..pos	Cer
	-15.629	-27.812	-12.183	0.000	0.011	CE.21.2.48…NH4.C48H82O2..pos	CE
	-14.304	-27.812	-13.508	0.000	0.011	PS.pmg.28.3.34…H.C34H62N1O9P1..pos	PS
	-14.260	-27.812	-13.552	0.001	0.013	DAG.36.2.39…K.C39H72O5..pos	DAG
	1.010	-6.589	-7.599	0.001	0.014	MAG.20.2.23…NH4.C23H42O4..pos	MAG
	-14.987	-27.812	-12.825	0.001	0.017	CE.23.4.50…NH4.C50H82O2..pos	CE
	-14.873	-27.812	-12.939	0.001	0.019	DAG.42.9.45…NH4.C45H70O5..pos	DAG
	2.036	-11.602	-13.638	0.002	0.034	PS.pmg.42.6.48..HC48H84N1O9P1.0.neg	PS
**Pt 2449PT**	17.824	-9.988	-27.812	0.000	0.000	TAG.57.2.60…NH4.C60H112O6..pos	TAG
**Pt 2614**	-15.588	-27.812	-12.223	0.000	0.000	HexCer.42.2.48…H.C48H91N1O8.1.neg	HexCer
	14.597	-13.215	-27.812	0.000	0.000	MAG.18.2.21…H.C21H38O4.0.neg	MAG
	15.199	-12.613	-27.812	0.000	0.000	FA.20.4.20…NH4.C20H32O2.1.neg	FA
	17.347	-10.465	-27.812	0.000	0.000	DAG.40.4.43…NH4.C43H76O5.0.neg	DAG
	-16.594	-27.812	-11.218	0.000	0.000	SM.32.0.37…H.C37H77N2O6P1.13.neg	SM
	15.888	-11.924	-27.812	0.000	0.002	GT1.46.0.NA..M.H.2Na.3.NA.0.neg	GT1
	1.721	-9.969	-11.690	0.000	0.035	MAG.18.2.21…NH4.C21H38O4.1.neg	MAG

Supplementary Figure 4: Inhibition of FASN alters lipid metabolism in CRC cells and PDX tumors. (**A**) Control (sh-Control) and FASN knockdown (sh-FASN) cells were labeled with [U-^{13}C]-glucose and lipid fractions were extracted and analyzed using SIRM. Even: lipids in which glycerol is not labeled, but fatty acyl chains are. Odd: lipids with both glycerol and fatty acyl chains newly synthesized from ^{13}C-glucose. (**B–D**) To compare TVB treated tumors to Control for each lipid log-intensities were normalized by total intensity of the sample. Principal component analysis (PCA) and heat maps are shown for three patients. (**E**) Lipids with adjusted *p*-value ≤ 0.05 for each patient are shown.
